# Specialist mental health services in England in 2014: overview of funding, access and levels of care

**DOI:** 10.1186/s13033-015-0023-9

**Published:** 2015-08-30

**Authors:** Mary Docherty, Graham Thornicroft

**Affiliations:** Institute of Psychiatry, Psychology and Neuroscience, King’s College London, London, UK; South London and Maudsley NHS Foundation Trust, London, UK; Health Service and Population Research Department, Institute of Psychiatry, Psychology and Neuroscience, King’s College London, De Crespigny Park, London, SE5 8AF UK

**Keywords:** Mental health services, Mental health systems, Healthcare resources, Finances, Investment, Access, Coverage, Levels of care

## Abstract

**Background:**

Since the economic recession began in 2008 anecdotal reports suggest that mental health services in England have experienced disinvestment, but published data to test this proposition are few.

**Method:**

This paper presents information from a wider range of official, research and grey literature sources aiming to: (1) assess whether governmental investment in publically funded mental health services has declined since the start of the economic recession in 2008; (2) to assess whether relative changes in mental health service investment over this period were or were not similar to trends in national investment in services for people with physical disorders, and (3) to interpret these findings in terms of met and unmet population levels needs for mental health care.

**Results:**

The key findings are that: across England social service expenditure reductions have led to a decrease of 48 % in the number of people with mental illness who receive such care, while direct NHS expenditure was reduced in some local areas by up to 32 %.

**Conclusions:**

The results of this overview suggest that there have been substantial reductions in the resources dedicated to mental health treatment and care in England since 2008, that such reductions seem not to have been applied to physical health services, and that these findings appear to run counter to the government policy of ‘parity of esteem; for mental and physical healthcare.

## Background

This year sees the publication of the Chief Medical Officer’s (CMO) Annual Public Health Report for England—the topic for 2014 was Public Mental Health. This brings together the best evidence in the field, set within a contemporary policy context, and informs the CMO’s recommendations for the further development of mental health in England. Although one in four adults experience at least one diagnosable mental health problem in any year, there is emerging evidence that most people with mental disorders in England receive no relevant healthcare [1].

As part of the CMO Report, we were commissioned to write an overview of gaps in mental health service provision in England. The aim of this paper is to summarise these gaps in terms of funding, access, treatment and care. We draw upon all the available data on resources (and *dis*investment) in mental health services in England in recent years, in relation to population levels of need. We go on to consider whether recent cuts in NHS mental health services are comparable the resource levels available in acute/physical care, given the recent governmental legal commitment to ‘parity of esteem’.

Mental health services in England have been historically characterised by significant variations in service provision, quality of care, and acceptability to users [2]. The National Service Framework for Mental Health (NSFMH) for England imposed a standard models of care, and was substantially implemented through a financial incentives system, and led to improvements in the availability and quality of provision in mental health services in England [3]. In 2011 the Coalition Government’s mental health strategy for England (entitled ‘No Health without Mental Health’) [4] recognised the need for ongoing improvements in quality and provision. It set six key targets including improvements in safety, patient-centeredness, recovery and physical health. Yet this emerged at the same time as considerable structural change in the NHS, related to the ‘Nicholson Challenge’ to hold overall expenditure steady, and with substantial restructuring for commissioners and providers in the NHS, governed by the 2012 Health and Social Care Act.

In terms of the national mental health service context, about a quarter of people with depression and anxiety in England receive treatment, most often in primary care settings [5, 6], while the large major of specialist mental health care is provided by National Health Service (NHS) staff. At the same time there has been a trend over the last decade for governments to stimulate a ‘mixed economy’ of NHS, for-profit and non-for-profit service providers. Until 1999 the pattern of mental health services was largely determined by local planners. A national 10 year plan for England was introduced in 1999 that set a clear profile of community mental health services to be provided in each local district [7]. Since 2010 a greater degree of ‘localism’ has been encouraged by the subsequent national mental health plan, in which local service commissioners can purchase services on the basis of a local assessment of needs, and not on the basis of a nationally specified pattern of care [4]. Some of the most important system wide key performance indicators, such as a maximum waiting time of 18 weeks to be seen by a specialist after a referral from primary care, especially excluded mental health care from this requirement, until a policy change in 2015.

Mental health services have long been considered to be the poor relation to physical/acute care, often described as being subject to less investment or greater disinvestment in times of plenty or scarcity [8]. Yet to date hard data on this alleged disparity have been difficult to identify. Conflicting Governmental guidance has emerged. The Department of Health’s 2014 policy guide on according equal value to mental and physical health (so-called ‘Parity of Esteem’) [9] is an important step forward in principle. Conversely, the imposition of a ‘tariff deflator’ (i.e. resource reduction), a fifth higher for mental health than for acute healthcare appears to undermine the overall policy intention [10].

## Method

In this context the method used in conducting this overview paper was to identify all available sources of information on governmental mental health investment (both health and social services) in England over the last decade. The sources used were: government Budgetary Programme Data, online searches of MedLine, PubMed, Ovid, Department of Health policy documents, Freedom of Information request reports, charitable and other grey literature reports, data from experts in the field (using a snowballing technique to identify all possible sources of relevant material), and other material from internet search engines. We also received detailed time trend budget data from one mental health provider trust in England.

## Results and discussion

### Increasing demand for mental health services during the economic recession

**Fig. 1:**
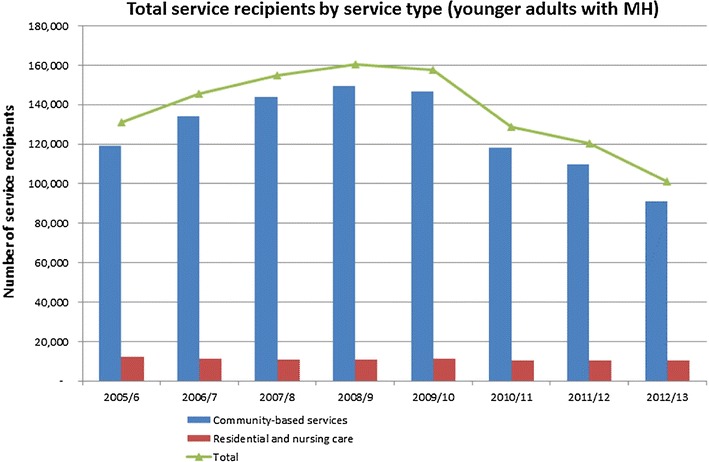
Total number of adults aged 18–64 with a mental health disorder receiving social care services by service type. Source [13]

**Fig. 2:**
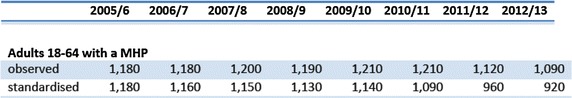
Observed and standardised net current expenditure by year (£millions). Source [13]

On the demand side, the prevalence of mental illness has been observed to increase during times of economic recession, while there is some evidence for increased suicidality during the period of economic austerity since 2008 [11, 12]. In terms of patterns of supply, one part of the picture is investment in social care support. Since 2005, 30,000 people with mental health problems have lost their social care support, following a £260 m (standardised figures) shortfall in funding due to cuts to local authority budgets, greater than for any other client group—a relative fall of 48 % of clients receiving social care [13] (see Figs. 1, 2).

### The ‘treatment gap’ in mental health service provision

Mental illnesses are the largest source of disability in the England. The Centre for Mental Health estimates that the aggregated economic and social costs of mental illness in England in 2009–2010 was £105.2 bn. This includes £21.3 bn in health and social care costs, £30.3 bn in lost economic output, and £53.6 bn in disability [14]. It is striking that in relation to the 28 % of total burden of disease which is attributable to mental illness in England, mental health care receives 13 % of total NHS spending [15]. There is an ‘inverse care law’ in that about 3/4 of people with long term physical disorders received treatment in England over the same time period, compared with about 1/4 of people with long term mental disorders. Indeed this is a global phenomenon—proportionately more people with physical disorders are treated in the poorest countries of the world, than are treated for mental disorders in high income countries such as England [1] (see Table 1).

**Table 1:** Treatment gap: treated prevalence for mental disorders in high, medium and low income settings

	High income countries (%)	Low and middle income countries
Physical disorders		
Diabetes	94	77
Heart disease	78	51
Asthma	65	44
Mental disorders		
Depression	29	8
Bipolar disorder	29	13
Panic disorder	33	9

Source: data from the 2002–2004 WHO World Mental Health Survey [1]

There is evidence that this ‘treatment gap’ is pervasive across all mental health disorders and all age groups (Box 1). In England, the most recent national psychiatric morbidity surveys for children and adolescents (2004) and adults (2007) show that the large majority of people with all mental disorders (except psychosis) receive no treatment [16, 17]. For example, a total of about 4.5 million adults and 525,000 children with anxiety/depression will not receive treatment [15, 16] this year. It is clear that very substantial change is required to achieve ‘parity of esteem’.

**Box 1:** Examples of gaps in treatment in UK provision of mental health services

Among people with severe mental illness, 29 % have received appropriate physical health checks in the past year [16] 20 % of adults who screened positive for Attention Deficit Hyperactivity Disorder were receiving medication, counselling or therapy for a mental health or emotional problem [16] 14 % of alcohol dependent adults were receiving treatment for a mental or emotional problem [16] Despite higher prevalence, older people are less likely than working age adults to be diagnosed with depression by their GP, and IAPT services are not yet configured to meet their needs, with IAPT access rates of just 5.2 % [16] Only 28 % of parents of children with a conduct disorder have sought advice from a mental health specialist [17] People with long-term conditions are 2–3 times more likely to experience mental illness than the general population, yet in 2/3 of cases depression goes undetected and untreated	

### Disinvestment and the treatment gap

These levels of treatment coverage (i.e. the proportion of all people with a condition who actually receive treatment) is largely related to investment in services (although some demand side factors, especially stigma, can play a major role [18]. There are now concerns that overall expenditure reductions will fail to meet the increasing demand and may compromise patient safety and service quality. Nevertheless, the primary source of data to examine such claims was recently decommissioned for funding by Government, so that such data are not available after 2012. Current information, including Programme Budgeting Data, do not provide sufficient detail to identify levels of need, or variations in service provision.

**Fig. 3:**
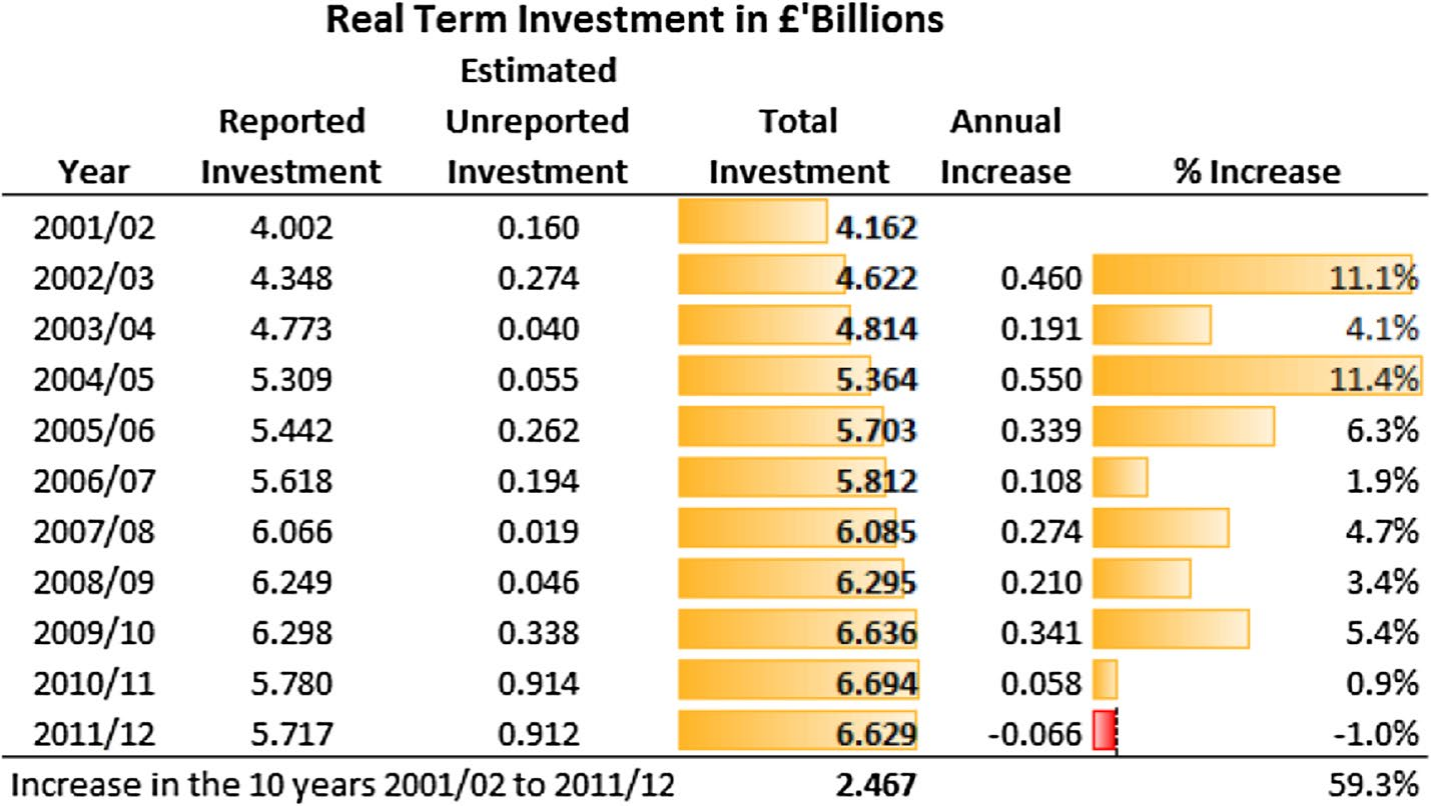
Total real investment in adult mental health services 2001/02 to 2011/12 (at 2011/12 pay and price levels). Source [34]

**Fig. 4:**
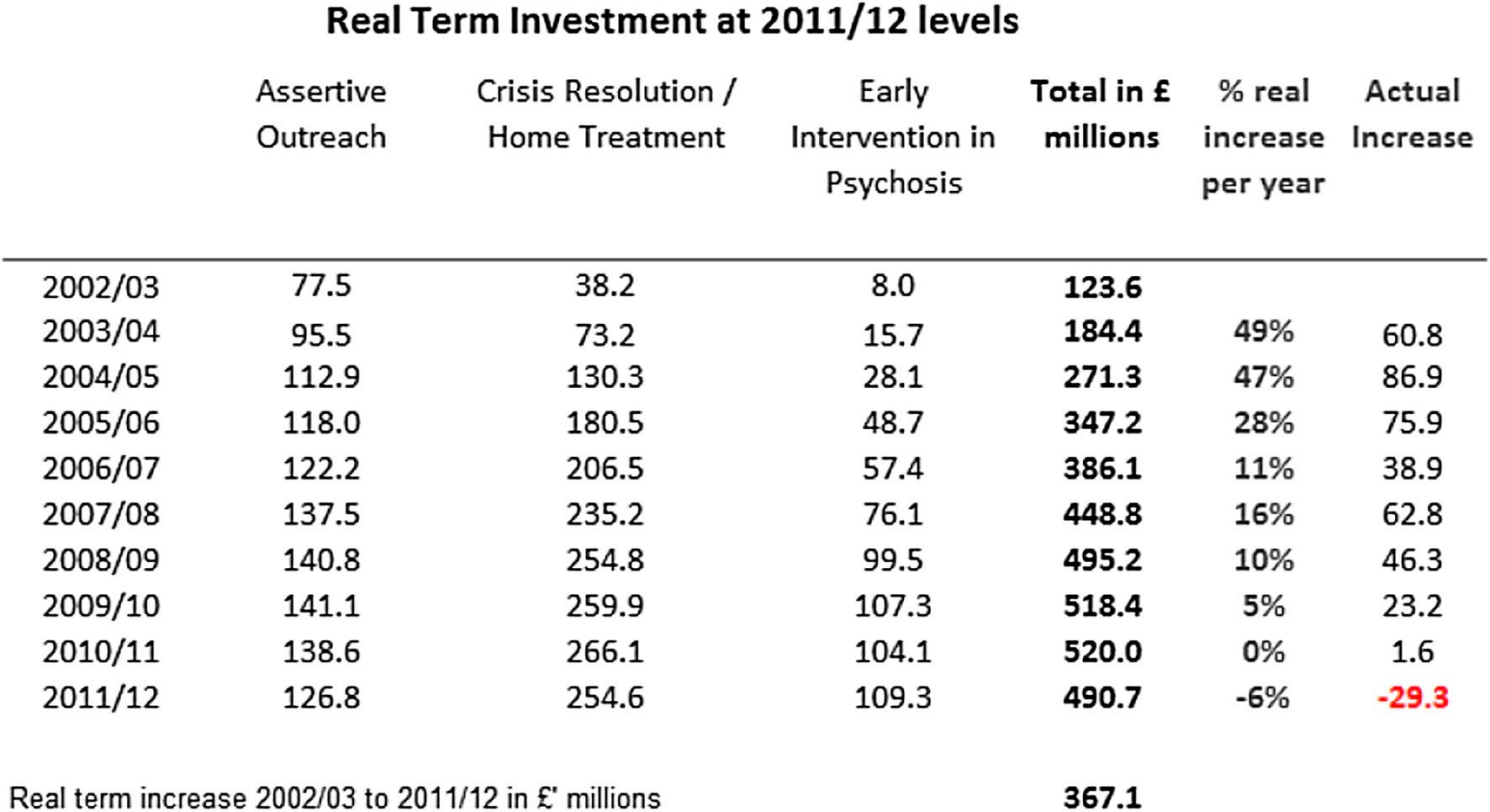
Real term investment in priority service areas 2002/3 to 2011/2. Source [34]

There is evidence that there have been reductions in investment in community mental health services in recent years (see Figs. 3, 4). The 2011/12 survey, for example, found that investment in mental health services for adults of working age (aged 18–64), to a total of £6.629 billion, fell in real terms from the previous year. This was the first real terms drop in investment since the survey began in 2001/02 [19]. Between 2010/11 and 2011/12 investment across the three priority areas in community mental health services (crisis resolution, early intervention and assertive outreach) fell, for the first time in a decade, by £29.3 million from £520 million to £490.7 million. This is significant given the evidence base for these services in reducing admissions to hospital and the continuing demand for acute beds [20]. Funding for older people’s mental health services was found to be under greater pressure, with a 1 % fall in resource allocation in the previous year to £2.830 billion in 2011/12. After allowing for inflation of service costs, this amounts to a real terms cut of 3.1 per cent. There is also evidence to suggest significant regional variations, for example service reductions being greater in London than in other parts of England [21].

There are no comparable official data for child and adolescent mental health services (CAMHS). We therefore gathered data by other means, including surveys by the third sector (i.e. both for profit and not for profit provider organisations) and Freedom of Information (FOI) requests. One survey found that 67 per cent of councils had reduced CAMHS funding between 2010 and 2013 [22]. Regional cuts in spending were as high as 12 % in the North East and 13 % in the East of England [22] over this period.

The BBC and the Community Care journal published figures in 2013, based upon a FOI request, with responses from 43 of 51 mental health trusts n England. Comparing 2011/12 budgets with those for 2013/14, they found a real terms reduction of 2.36 %, while funding for psychological therapies increased by 6 % in real terms (source: http://www.bbc.com/news/health-24537304.)

Despite official figures estimating 1–2 % real terms decreases in expenditure, case study information from individual Trusts published in the Chief Medical Officer’s 2014 Report suggests that these figures may fall far short of actual disinvestment. One large metropolitan mental health trust reported that over the period from 2009/10 to 2013/14 it had seen a net reduction in funding of £12 m, ‘with the pace of net disinvestment being accelerated and set to do so further’. They report that ‘If the funding provided by the Department of Health and which passes through Clinical Commissioning Groups (CCGs) then the net gain to local CCGs from disinvestment and efficiency in local mental health services has been approximately £50 m. In each of our local CCGs we have seen net savings from mental health services of at least 32 % over the last 7 years.’ These are far in excess of official estimates, and support a series of concerns summarised in Box 2.

**Box 2:** Key messages on the treatment gap in mental health services in England

There is a very significant overall *treatment gap* in mental health with about 75 % of people with mental illness receiving no treatment at all [1] The treatment gap contributes to unacceptably high *mortality rates*, as the available data suggest that people with mental illness can die up to 15–20 years earlier on average than individuals without mental illness [35, 36] There are *significant and inappropriate variations* in the delivery of mental health services [9] Information on mental health service expenditure currently lack sufficient detail There is a clear fall in investment and expenditure despite evidence of an increase in mental health burden [12, 17, 21] It is unclear whether the disinvestment has been greater for mental than for physical health provision There appear to be considerable discrepancies between overall national figures for resource reductions and the figures available locally from mental health Trusts There are no available data sets which capture the implications for mental health services of aggregate expenditure reductions across multiple sectors (criminal justice, social care, non-statutory, and the voluntary sectors)	

### Service accessibility and waiting times

Mental health services are currently exempt from the 18 week maximum waiting time for service access stipulated in the NHS Constitution. Service user data indicates that over 12 % of people wait longer than 1 year to start treatment, whilst 54 % wait over 3 months [23]. The number of people presenting in a mental health crisis have increased in recent years [24], and 40 % of mental health trusts have staffing levels below established benchmarks for crisis services (http://www.mind.org.ug/crisiscare). Waiting times for emergency assessment, for example in police cells or Mental Health Act Section 136 Suites, have escalated in recent years, along with increasing use of the police and criminal justice system to ‘care’ for individuals when unwell due to insufficient capacity in mental health services [25]. Nevertheless the quality of data about these acute issues is poor. The Care Quality Commission, for example, has raised concerns about bed occupancy rates for many years [26] yet their most recent report does not report on bed occupancy rates (see: http://www.england.nhs.uk/statistics/statistical-work-areas/bed-availability-and-occupancy/bed-data-overnight/).

Limitations in data sources have led to increased freedom of information requests and professional surveys to investigate these concerns (Box 3). These findings include a reported 9 % reduction in mental health beds between 2011 and 2012 and a doubling of patients being sent out of area for treatment between 2011/12 and 2013/4 [27] (see: http://www.communitycare.co.uk/2013/10/16/patients-at-risk-as-unsafe-mental-health-services-reach-crisis-point-2/#.U3WoVq1dUv0)

**Box 3:** Summary of recent freedom of information requests and survey findings

A minimum of 1711 mental health beds have been closed since April 2011, including 277 between April and August 2013. This is a 9 % reduction in the total number of mental health beds—18,924—available in 2011/12. http://www.communitycare.co.uk/2013/10/16/patients-at-risk-as-unsafe-mental-health-services-reach-crisis-point-2/#.U3WoVq1dUv0	
A 2013 survey of members of the Child and Adolescent Psychiatry Faculty was carried out 77 % of respondents to a 2013 survey of members of the Child and Adolescent Psychiatry Faculty about their experience of admitting young people to inpatient unit reported difficulties in accessing admissions to inpatient beds. 79.1 % respondents reported safeguarding concerns/incidents whilst waiting for a bed; 76.5 % reported young people with unacceptably high risk profiles being managed in the community due to lack of beds; 61.9 % reported young people being held in inappropriate settings [37]	
Freedom of information data from 30 trusts, reported the number of patients sent out of area has more than doubled between 2011/2 and 2013/14 (1301 in 2011/12 to 3024 in 2012/3). The costs associated with this reported by 23 Trusts show an increase in expenditure from £21.1 m in 2011/12 to £38.3 m in 2012/13 http://www.bbc.co.uk/news/uk-27285555	

### Admission, compulsion and suicides

The number of psychiatric hospital admissions is now about double that figure for 2000 [19]. Use of the Mental Health Act has also steadily grown in recent years, and in 2012/13 there was a 4 per cent increase in compulsory detentions in comparison to the previous year [28]. The Care Quality Commission and service user experience surveys show ongoing poor involvement of service users in their care [29]. The Care Quality Commission reports ongoing inappropriate restrictive practices and cultures in many wards demonstrating a ‘significant gap between practice and the ambitions of the national mental health policy [30].

## Discussion

Disregard for the needs of people with mental illness has been described by some authors as ‘structural discrimination’ [31, 32]. This concept can also be applied to lack of investment in information infrastructure to be able to know whether services are improving or not.

There are several important limitations of this study. The research deliberately sought all relevant sources of information about the levels of investment in mental health care in England, and recent trends, and this meant that these sources were very heterogeneous and drew upon a wide variety of official data, research reports, the grey literature and case studies. We therefore would not place very heavy weight upon individual sources, but rather wish to interpret the overall pattern of results. Second, the time frame used for the data sources varied somewhat, with some referring to the period since 2008 when the economic recession began, and other to the period of the government at the time, which came into power in 2010. Further, we have brought together information across a wide range of sources, but it is true that there are few sources of information about true prevalence and treated prevalence across all diagnostic groups, and such data are not routinely and repeatedly collected and reported by the government. It also needs to be kept in mind that rates of service utilization (and deductions about rates of unmet need) may differ when reported by service users or by service providers [33, 34]. In addition, it is possible that there were types of substitution (for example with fewer community services in recent years has this been associated with a greater demand for psychiatric beds?), but we were not able to identify data to bear upon this issue.

The recent governmental commitment to ‘parity of esteem’ [9] is long overdue. Yet the policy requirements which have been applied to acute/physical healthcare, such as the 18 week waiting time limit, have still not been applied equally to mental health care. It is also clear that unintended consequences of the tariff system (cut more in recent years for mental than for mental health care) have systematically disadvantaged both commissioners and providers of mental health care. Poorly integrated financial monitoring processes have contributed to a failure to alert all parts of the NHS to how far resource reductions have harmed the quantity and quality of mental health care in recent years. In the post 2013 structure of the NHS separate health, social care and public health outcomes frameworks are making it even harder to commission joint or integrated services, to avoid gaps in provision, and to monitor progress or deterioration in services. At the same time it needs to be acknowledged that within this context of overall disinvestment in mental health care, some services are being expanded, particularly the remit of the Improving Access to Psychological Therapies (IAPT) services. Taken as a whole, these findings are far from reassuring for everyone dedicated to better mental health care in England.
